# Predictors of Prolonged ICU Stay After Isolated CABG: The Role of MiECC

**DOI:** 10.3390/medicina62071289

**Published:** 2026-07-03

**Authors:** Alper Özbakkaloğlu, Ömer Faruk Rahman, Mert Arslangilay, Ercan Keleş, Önder Turgut Bozkurt, Dağlar Cansu, Şahin Bozok

**Affiliations:** Department of Cardiovascular Surgery, İzmir Bakırçay University, Menemen 35665, Turkey; alper.ozbakkaloglu@bakircay.edu.tr (A.Ö.); marslangilay@gmail.com (M.A.); mevertra@yahoo.co.uk (E.K.); drotb2002@hotmail.com (Ö.T.B.); daglarcansu@icloud.com (D.C.); sahinboz@yahoo.com (Ş.B.)

**Keywords:** coronary artery bypass grafting, prolonged ICU stay, minimally invasive extracorporeal circulation, ejection fraction, leukocyte count

## Abstract

*Background and Objectives*: Prolonged intensive care unit (ICU) stay following coronary artery bypass grafting (CABG) remains a major clinical challenge. The aim of this study was to identify preoperative and intraoperative factors that may predict prolonged ICU requirement in patients undergoing isolated CABG and to evaluate the impact of minimally invasive extracorporeal circulation (MiECC) utilization on this outcome. *Materials and Methods*: Patients who underwent isolated CABG between January 2024 and April 2025 were retrospectively analyzed. Prolonged ICU stay was defined as a postoperative intensive care duration exceeding 72 h. Predictors of prolonged ICU stay were evaluated using univariate logistic regression and four multivariable logistic regression models. Receiver operating characteristic (ROC) curve analyses were performed to assess the discriminative performance of significant and clinically relevant variables as well as the multivariable models. *Results*: A total of 82 patients were included and stratified into a prolonged ICU stay group (n = 32) and a non-prolonged ICU stay group (n = 50). The preoperative left ventricular ejection fraction (LVEF) was significantly lower (52.5% vs. 60%, *p* = 0.012) and preoperative leukocyte counts significantly elevated (8.4 vs. 7.97 × 10^3^/µL, *p* = 0.049) in the prolonged stay group. MiECC was employed in 28.1% of patients with a prolonged ICU stay and 30% of those with a non-prolonged stay (*p* = 0.530). Across four multivariable logistic regression models, lower preoperative ejection fraction and higher preoperative leukocyte count were identified as independent predictors of prolonged ICU stay, whereas MiECC utilization was not independently associated with this outcome. *Conclusions*: Lower preoperative ejection fraction and higher baseline leukocyte count were independently associated with prolonged ICU stay. No independent association between MiECC utilization and prolonged ICU stay could be demonstrated. These findings suggest that preoperative cardiac function and inflammatory status may contribute to ICU requirements following isolated CABG. Further prospective studies are warranted to validate these findings.

## 1. Introduction

Coronary artery bypass grafting (CABG) remains the preferred revascularization strategy for complex multivessel coronary artery disease [1]. Although advances in surgical techniques and perioperative care have significantly improved clinical outcomes, prolonged intensive care unit (ICU) stay continues to be a major clinical challenge. Prolonged ICU stay following isolated CABG surgery is intricately associated with severe postoperative complications, including nosocomial infections, multi-organ dysfunction, and prolonged ventilator dependency [2,3]. Consequently, extended ICU durations not only adversely affect patient prognosis and increase mortality rates, but also impose a substantial financial and logistical burden on healthcare systems worldwide [4]. Identifying patients at risk for extended ICU admission before they enter the operating room is crucial for optimizing resource allocation and tailoring perioperative management. Previous studies have identified several preoperative predictors of prolonged ICU stay, such as advanced age, low left ventricular ejection fraction, renal impairment, and existing comorbidities like diabetes mellitus and chronic obstructive pulmonary disease [2]. In recent years, laboratory-derived inflammatory and hematological parameters, including composite inflammatory indices, have also emerged as practical and readily available predictors of adverse postoperative outcomes following cardiac surgery [5,6,7]. However, while these patient-related risk factors are non-modifiable, the choice of extracorporeal circulation strategy represents a critical modifiable intraoperative factor that could significantly influence the postoperative course. Conventional cardiopulmonary bypass (cCPB) is known to trigger a robust systemic inflammatory response syndrome (SIRS), leading to hemodilution, coagulopathy, and subsequent end-organ damage, all of which are catalysts for a prolonged ICU stay [8]. To minimize these effects, minimally invasive extracorporeal circulation (MiECC) systems have been developed. By employing closed circuits, significantly reducing priming volumes, and minimizing blood–air contact, MiECC aims to preserve physiological homeostasis, attenuate systemic inflammation, and reduce the need for blood transfusions [9,10,11]. The aim of this study was to identify preoperative and intraoperative factors that may predict prolonged ICU stay in patients undergoing isolated CABG and to evaluate the impact of MiECC utilization on this outcome.

## 2. Materials and Methods

### 2.1. Ethical Approval

The study protocol was approved by the Non-Interventional Clinical Research Ethics Committee of İzmir Bakırçay University (Decision No: 2375, Research No: 2363, Date: 14 August 2025). The study was conducted in accordance with the principles of the Declaration of Helsinki.

### 2.2. Patient Selection and Study Design

**Figure 1:**
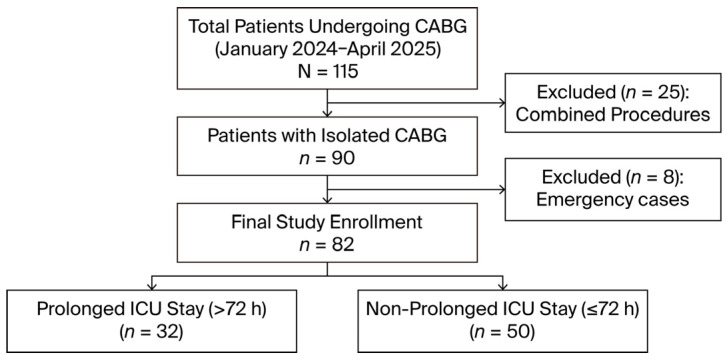
Flowchart of patient selection and study group allocation.

This retrospective observational study included patients who underwent isolated coronary artery bypass grafting (CABG) between January 2024 and April 2025. Emergency cases and patients with incomplete clinical data were excluded from the study. The patient inclusion and exclusion process is illustrated in Figure 1.

Data regarding demographic characteristics, comorbidities, preoperative laboratory findings, operative variables, and postoperative intensive care unit (ICU) stay durations were obtained from electronic medical records. Preoperative laboratory parameters were collected from the most recent blood tests performed within one week prior to surgery. Left ventricular ejection fraction (LVEF) values were obtained from the most recent transthoracic echocardiographic examination performed at hospital admission before surgery. All echocardiographic assessments were performed and reported by experienced cardiologists as part of routine clinical practice. In addition, data regarding new-onset atrial fibrillation, acute kidney injury, reoperation, and in-hospital mortality were retrieved from electronic medical records. Acute kidney injury was defined according to the Kidney Disease: Improving Global Outcomes (KDIGO) 2012 criteria as an increase in serum creatinine of ≥0.3 mg/dL within 48 h or ≥1.5 times the baseline value within 7 days [12]. ICU stay duration was recorded in hours for all patients and subsequently categorized according to a predefined threshold. Prolonged ICU stay was defined as a postoperative ICU stay exceeding 72 h, consistent with previous studies evaluating prolonged ICU admission after cardiac surgery [13,14].

### 2.3. Surgical Technique and Perfusion Strategy

All surgical procedures were performed through median sternotomy under cardiopulmonary bypass support by the same experienced surgical team.

In the conventional cardiopulmonary bypass group, standard open-circuit extracorporeal circulation systems were utilized, and activated clotting time (ACT) was maintained above 480 s throughout bypass.

In the MiECC group, a type IV minimally invasive extracorporeal circulation system characterized by a closed-circuit design, reduced priming volume, and minimized blood–air interaction was used. According to institutional perfusion protocols, lower ACT targets were employed in the MiECC group, and ACT was maintained above 350 s.

The choice between conventional cardiopulmonary bypass and MiECC was not based on a standardized patient selection protocol but was made individually according to the surgeon’s clinical judgment and the prevailing technical and operational conditions. As MiECC requires specialized equipment and appropriate circuit availability, it was used in cases where these requirements could be met.

### 2.4. Statistical Analysis

Statistical analyses were performed using SPSS version 23.0 (IBM Corp., Armonk, NY, USA). Continuous variables were tested for normality. Variables with normal distribution were expressed as mean ± standard deviation (SD), whereas non-normally distributed variables were presented as median and interquartile range (IQR).

Comparisons between groups were performed using the Chi-square test, Student’s *t*-test, or Mann–Whitney U test, as appropriate. Predictors of prolonged ICU stay were evaluated using univariate and several multivariable logistic regression models. To assess the discriminative performance of statistically significant and clinically relevant variables, as well as the multivariable models, receiver operating characteristic (ROC) curve analyses were performed and the area under the curve (AUC) was calculated. A *p*-value of <0.05 was considered statistically significant.

## 3. Results

A total of 82 patients who underwent isolated CABG were included in the study. Patients were stratified into two groups based on the duration of their intensive care unit admission: the prolonged ICU stay group (n = 32) and the non-prolonged ICU stay group (n = 50). Among patients without prolonged ICU stay, the mean ICU stay duration was 53.16 ± 11.95 h and the median was 53 h (IQR: 7). In patients with prolonged ICU stay, the mean ICU stay duration was 105.53 ± 35.80 h and the median was 99 h (IQR: 44). In the overall study population, the mean ICU stay duration was 73.60 ± 35.18 h and the median was 59.5 h (IQR: 40). The mean age was 64 ± 11.2 years in the prolonged group and 63.75 ± 9.08 years in the non-prolonged group (*p* = 0.805). There were no statistically significant differences regarding gender distribution, body surface area (BSA), or the incidence of underlying conditions such as diabetes mellitus, hypertension, hyperlipidemia, chronic obstructive pulmonary disease (COPD), and chronic kidney disease (all *p* > 0.05).

An analysis of the preoperative diagnostic parameters revealed distinct variations between the two groups. Preoperative LVEF was significantly lower in patients who subsequently required prolonged ICU care compared to those with a standard ICU duration (52.5 vs. 60, *p* = 0.012). Furthermore, the prolonged ICU stay group exhibited significantly elevated preoperative leukocyte counts (8.4 vs. 7.97 10^3^/µL, *p* = 0.049). Other routine preoperative laboratory indices, including hemoglobin, hematocrit, renal function tests (urea, creatinine), liver enzymes (AST, ALT), electrolyte panels, albumin, C-reactive protein (CRP), and HbA1c levels, demonstrated no statistically significant differences between the groups (*p* > 0.05 for all) (Table 1).

The intraoperative characteristics of the study cohort are summarized in Table 2.

The type of cardiopulmonary bypass (CPB) utilized did not significantly differ between the groups; MiECC was employed in 28.1% (n = 9) of patients with a prolonged ICU stay and 30% (n = 15) of those with a non-prolonged stay (*p* = 0.530). Although the mean cross-clamp time (75 vs. 60 min, *p* = 0.099), total CPB time (98 vs. 93 min, *p* = 0.161), and average number of bypass grafts (3.5 vs. 3, *p* = 0.173) were slightly higher in the prolonged ICU stay group, none of these intraoperative variables reached statistical significance.

Postoperative outcomes were also evaluated. New-onset atrial fibrillation was observed in 13 patients (26.0%) in the non-prolonged ICU stay group and in 12 patients (37.5%) in the prolonged ICU stay group, with no statistically significant difference between the groups (*p* = 0.270). Acute kidney injury occurred in eight patients (16.0%) and six patients (18.8%), respectively (*p* = 0.747). Reoperation was required in one patient in each group, corresponding to rates of 2.0% and 3.1%, respectively (*p* = 1.000). In-hospital mortality was observed in three patients (6.0%) in the non-prolonged ICU stay group and in one patient (3.1%) in the prolonged ICU stay group, with no statistically significant difference between the groups (*p* = 1.000).

In the univariate logistic regression analysis, an increase in preoperative ejection fraction was associated with a reduced likelihood of prolonged ICU stay (OR = 0.935, 95% CI: 0.889–0.984, *p* = 0.010). In contrast, higher preoperative leukocyte counts were significantly associated with an increased risk of prolonged ICU stay (OR = 1.382, 95% CI: 1.092–1.749, *p* = 0.007). Cross-clamp time demonstrated a borderline association with prolonged ICU stay (OR = 1.013, 95% CI: 0.999–1.026, *p* = 0.064). New-onset atrial fibrillation (OR = 1.708, 95% CI: 0.657–4.437, *p* = 0.272) and acute kidney injury (OR = 1.212, 95% CI: 0.378–3.888, *p* = 0.747) were not significantly associated with prolonged ICU stay. Similarly, age (OR = 1.003, 95% CI: 0.959–1.050, *p* = 0.885), diabetes mellitus (OR = 1.273, 95% CI: 0.506–3.204, *p* = 0.609), COPD (OR = 1.600, 95% CI: 0.214–11.969, *p* = 0.647), cardiopulmonary bypass duration (OR = 1.008, 95% CI: 0.998–1.018, *p* = 0.122), number of bypass grafts (OR = 1.385, 95% CI: 0.900–2.133, *p* = 0.139), CRP level (OR = 0.998, 95% CI: 0.983–1.013, *p* = 0.788), and MiECC utilization (OR = 0.913, 95% CI: 0.343–2.432, *p* = 0.856) were not significantly associated with prolonged ICU stay (Table 3).

Four different multivariate logistic regression models were constructed to identify independent predictors of prolonged ICU stay. The variables found to be significant in the univariate analysis, together with MiECC utilization due to its clinical relevance, were evaluated in different model combinations, and the results are presented in Table 4.

Model 1 included age, ejection fraction (EF), white blood cell count (WBC), and MiECC utilization. The model was statistically significant overall (χ^2^ = 14.317, *p* = 0.006) and explained 21.7% of the variance in prolonged ICU stay according to the Nagelkerke R^2^ value. In this model, each unit increase in EF was associated with a lower likelihood of prolonged ICU stay (OR = 0.938, 95% CI: 0.889–0.990, *p* = 0.021). In contrast, each unit increase in WBC was associated with a higher likelihood of prolonged ICU stay (OR = 1.351, 95% CI: 1.052–1.733, *p* = 0.018). Age (OR = 1.015, 95% CI: 0.963–1.069, *p* = 0.587) and MiECC utilization (OR = 0.727, 95% CI: 0.242–2.187, *p* = 0.570) were not independently associated with prolonged ICU stay.

Model 2 additionally included cross-clamp time. The model remained statistically significant (χ^2^ = 16.468, *p* = 0.006) and showed a slightly improved explanatory capacity compared with Model 1 (Nagelkerke R^2^ = 0.247). WBC remained independently associated with prolonged ICU stay (OR = 1.359, 95% CI: 1.054–1.752, *p* = 0.018), whereas the association of EF was attenuated and remained at borderline significance (OR = 0.948, 95% CI: 0.897–1.002, *p* = 0.058). Cross-clamp time was not independently associated with prolonged ICU stay (OR = 1.010, 95% CI: 0.995–1.026, *p* = 0.175). Age (OR = 1.022, 95% CI: 0.968–1.079, *p* = 0.433) and MiECC utilization (OR = 0.839, 95% CI: 0.275–2.563, *p* = 0.758) were also not independently associated with prolonged ICU stay.

Model 3 included cardiopulmonary bypass duration in addition to the variables in Model 1. The model was statistically significant overall (χ^2^ = 15.609, *p* = 0.008), with a Nagelkerke R^2^ value of 0.235. In this model, EF remained independently and inversely associated with prolonged ICU stay (OR = 0.944, 95% CI: 0.894–0.997, *p* = 0.038), whereas WBC remained independently and positively associated with prolonged ICU stay (OR = 1.361, 95% CI: 1.055–1.757, *p* = 0.018). Cardiopulmonary bypass duration was not an independent predictor of prolonged ICU stay (OR = 1.006, 95% CI: 0.995–1.017, *p* = 0.255). Age (OR = 1.020, 95% CI: 0.967–1.077, *p* = 0.464) and MiECC utilization (OR = 0.813, 95% CI: 0.267–2.481, *p* = 0.716) were not associated with prolonged ICU stay.

Model 4 included age, EF, CRP, and MiECC utilization. This model did not reach overall statistical significance (χ^2^ = 8.276, *p* = 0.082). Although EF was significantly associated with prolonged ICU stay within the model (OR = 0.928, 95% CI: 0.879–0.980, *p* = 0.007), CRP level (OR = 0.995, 95% CI: 0.978–1.011, *p* = 0.526), age (OR = 1.008, 95% CI: 0.960–1.058, *p* = 0.752), and MiECC utilization (OR = 0.683, 95% CI: 0.233–1.997, *p* = 0.486) were not significantly associated with prolonged ICU stay.

**Figure 2:**
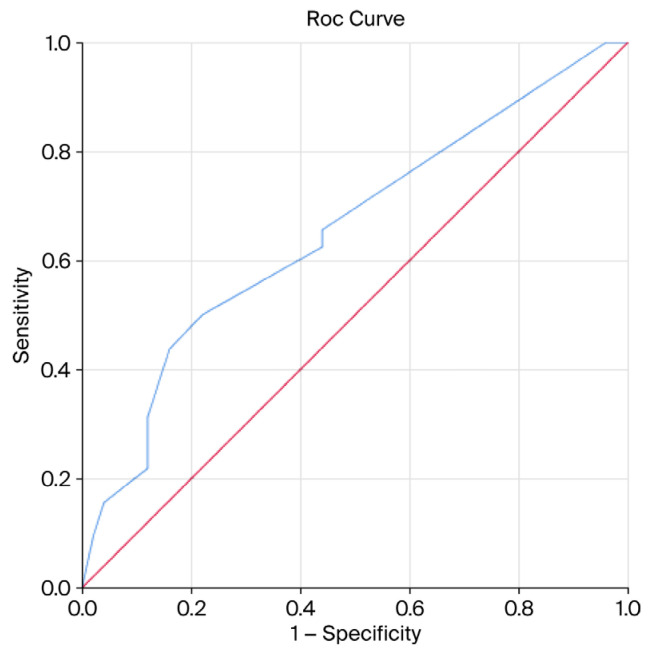
Receiver operating characteristic (ROC) curve analysis of preoperative ejection fraction for predicting prolonged ICU stay following isolated CABG.

**Figure 3:**
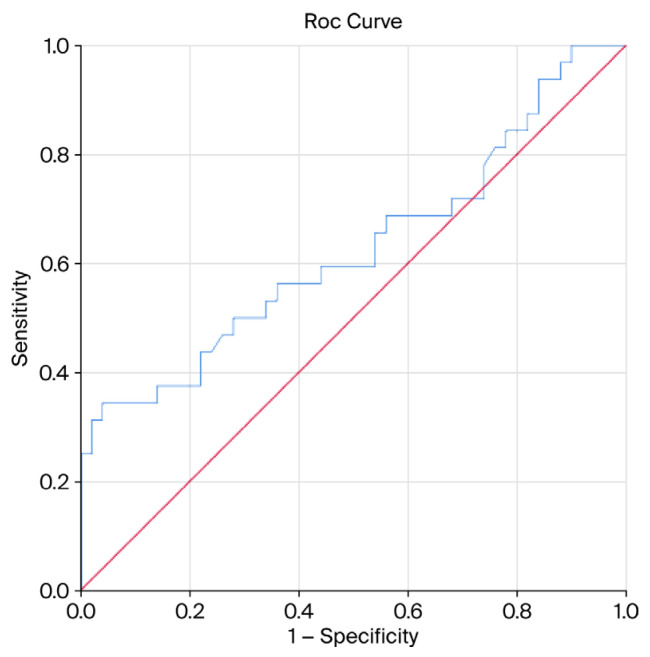
Receiver operating characteristic (ROC) curve analysis of preoperative white blood cell count for predicting prolonged ICU stay following isolated CABG.

ROC analyses were performed to evaluate the discriminative performance of both individual predictors and multivariate logistic regression models for prolonged ICU stay (Table 5). In the univariate analyses, the AUC for EF was 0.656 (95% CI: 0.533–0.779; *p* = 0.018) (Figure 2). The optimal cutoff value for EF was ≤59.0, yielding a sensitivity of 65.6% and a specificity of 56%. The AUC for WBC was 0.627 (95% CI: 0.496–0.757; *p* = 0.054), with an optimal cutoff value of ≥8.25 × 10^3^/µL (Figure 3). At this threshold, the sensitivity and specificity were 56.3% and 64%, respectively.

**Figure 4:**
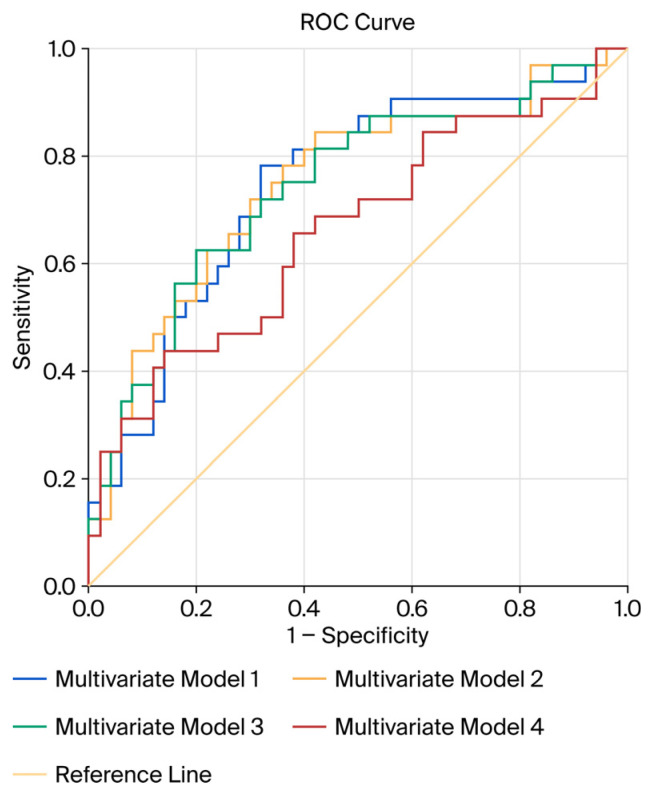
Receiver operating characteristic (ROC) curves of the four multivariate logistic regression models for predicting prolonged ICU stay following isolated CABG.

In the ROC analyses of the multivariate models, the AUC values for Model 1, Model 2, and Model 3 were 0.745, 0.749, and 0.743, respectively, and all models demonstrated statistically significant discriminative performance (all *p* < 0.001). Sensitivity was 84.4% across these models, whereas specificity ranged from 48% to 56%. The highest AUC was observed for Model 2 (AUC = 0.749, 95% CI: 0.637–0.862). In Model 4, in which CRP was evaluated instead of WBC, the AUC was 0.662 (95% CI: 0.537–0.786; *p* = 0.014), with a sensitivity of 71.9% and a specificity of 50%. The ROC curves of the multivariate models are presented in Figure 4.

## 4. Discussion

In this study, factors associated with prolonged ICU stay following isolated CABG were evaluated, and the potential impact of MiECC utilization on this outcome was investigated. Our findings demonstrated that lower preoperative ejection fraction and higher baseline leukocyte count were the most consistent independent predictors of prolonged ICU stay. These findings were further supported by additional multivariable models and ROC analyses. In contrast, an independent association between MiECC utilization and prolonged ICU stay could not be demonstrated in either the univariate or multivariable analyses.

The association between reduced preoperative LVEF and extended ICU duration is well supported by the existing literature [15]. A diminished ejection fraction is a direct indicator of compromised baseline cardiac reserve. Following the myocardial stress induced by ischemia–reperfusion injury during CABG, patients with low LVEF are significantly more susceptible to developing low cardiac output syndrome (LCOS) postoperatively [16]. Consequently, these patients often require prolonged inotropic support, extended mechanical ventilation, and more intensive hemodynamic monitoring, all of which necessitate an extended ICU stay [17]. Our multivariable analyses further corroborated that baseline myocardial function remains a cornerstone determinant of early postoperative recovery, with lower preoperative ejection fraction remaining independently associated with prolonged ICU stay in most of the evaluated models.

Furthermore, our study identified elevated preoperative leukocyte counts as an independent predictor of prolonged ICU stay. While routine preoperative laboratory values are often overlooked if they fall within borderline ranges, a higher baseline leukocyte count indicates a preexisting state of subclinical systemic inflammation [18]. When a patient with a preexisting proinflammatory state undergoes surgical trauma and extracorporeal circulation, they are more prone to an exaggerated systemic inflammatory response syndrome (SIRS) postoperatively [19]. This hyperinflammatory state can lead to endothelial dysfunction, fluid extravasation, and transient organ dysfunction, subsequently delaying extubation and ICU discharge. Although the absolute difference in leukocyte counts between the groups was relatively modest, leukocyte count remained independently associated with prolonged ICU stay across all statistically significant multivariable models. The ROC analysis identified a threshold of 8.25 × 10^3^/µL; however, its discriminative performance was modest, indicating that leukocyte count has limited predictive value as a standalone marker. Therefore, it should not be regarded as a strong standalone prognostic marker but rather as a complementary biomarker that may support clinical assessment when considered together with other clinical and laboratory findings. Notably, CRP did not demonstrate an independent association with prolonged ICU stay, and the model incorporating CRP failed to achieve overall statistical significance. These findings suggest that preoperative leukocytosis may be more closely associated with prolonged ICU stay than CRP in this setting.

One of the principal findings of our study was the absence of a significant association between MiECC utilization and reduced ICU duration. MiECC systems are explicitly designed to attenuate SIRS, reduce hemodilution, and minimize microembolic events by minimizing blood–air contact and eliminating venous reservoirs [9,10,11]. Although several studies have reported that MiECC facilitates faster postoperative recovery [10,20], our results suggest a different paradigm for ICU stay. We hypothesize that the physiological impact of a patient’s preoperative risk profile, specifically baseline cardiac reserve and inflammatory status, outweighs the intraoperative benefits of the perfusion system. In other words, a refined surgical circuit cannot completely negate the high-risk trajectory established by compromised preoperative hemodynamics and inflammation. Additionally, standardized institutional ICU weaning and discharge protocols may have contributed to similar ICU durations between the two perfusion groups. Furthermore, MiECC utilization was not independently associated with prolonged ICU stay in any of the multivariable models. Although the odds ratios for MiECC were consistently below 1, the confidence intervals were wide and included the null value. Therefore, while a substantial independent effect could not be demonstrated in the present cohort, a modest protective effect cannot be definitively excluded.

Previous studies have reported associations among postoperative atrial fibrillation, acute kidney injury, and prolonged ICU stay after CABG [21,22]. However, no significant associations were observed in the present study. Notably, a recent study reported that postoperative atrial fibrillation lasting ≥48 h, rather than postoperative atrial fibrillation per se, was independently associated with prolonged ICU stay, whereas postoperative atrial fibrillation was only analyzed as a dichotomous variable in our study [23]. Similarly, the relatively low incidence of postoperative acute kidney injury in the present cohort may also have limited the statistical power to detect a significant association. Together with the limited number of prolonged ICU stay events, these factors may have reduced the ability to detect statistically significant associations. Therefore, these findings should not be interpreted as evidence that postoperative atrial fibrillation or acute kidney injury have no impact on ICU stay.

ROC analyses demonstrated that the discriminative performance of the multivariable models was superior to that of the individual predictors. While ejection fraction and leukocyte count showed only modest predictive ability when evaluated separately, the multivariable models achieved AUC values ranging from 0.743 to 0.749. Moreover, the addition of cross-clamp time or cardiopulmonary bypass duration did not meaningfully improve model performance. These findings suggest that the predictive contribution of these intraoperative time-related variables may be limited within the context of the present study. Although the multivariable models were statistically significant, the relatively low Nagelkerke R^2^ values (21.7–24.7%) indicate that only a limited proportion of the variability in prolonged ICU stay was explained by the models. This finding suggests that prolonged ICU stay is a multifactorial outcome influenced not only by preoperative characteristics, but also by intraoperative factors, postoperative clinical course, and other unmeasured variables.

Several limitations of this study should be acknowledged. First, the study has a retrospective and single-center design, which increases the risk of selection bias and limits the generalizability of the findings. Second, the relatively small sample size may have affected the statistical power required to detect subtle differences between the MiECC and conventional cardiopulmonary bypass groups. In particular, the limited number of patients undergoing MiECC and the relatively low number of prolonged ICU stay events may have reduced the ability to detect small or moderate effect sizes, increasing the possibility of a type II error. Furthermore, the confidence intervals around the MiECC effect estimates were relatively wide, indicating substantial uncertainty regarding the magnitude of the observed associations. Moreover, the limited number of prolonged ICU stay events relative to the number of variables evaluated in the multivariable models resulted in relatively low events-per-variable (EPV) ratios, which may have affected model stability and increased the risk of overfitting. In addition, although clinically relevant variables such as diabetes mellitus, chronic kidney disease, COPD, and the number of bypass grafts were evaluated, they were not incorporated into the multivariable models because of the limited number of prolonged ICU stay events and the need to minimize the risk of overfitting. None of these variables showed a statistically significant association with prolonged ICU stay in the univariate analyses. Nevertheless, residual confounding related to clinically relevant variables that could not be included in the final models cannot be completely excluded, and the reported independent associations should therefore be interpreted with appropriate caution.

In addition, the absence of a standardized protocol for allocation to MiECC may have introduced selection bias. There is also no universally accepted definition of prolonged ICU stay in the cardiac surgery literature, and different studies have used varying thresholds ranging from 24 h to 14 days [15]. Although the 72 h threshold used in the present study was based on previous cardiac surgery studies, this heterogeneity may affect comparisons between studies [13,14]. Moreover, the assessment of systemic inflammation was limited to routine leukocyte counts, and the inclusion of more specific inflammatory biomarkers could have provided additional mechanistic insight.

The use of a single preoperative blood measurement represents another limitation of the study. In addition, data regarding preoperative medication use, which could potentially influence both systemic inflammatory response and baseline hemodynamic status, were not available. Moreover, due to the retrospective nature of the study, data regarding mechanical ventilation duration and postoperative infectious complications were not consistently and reliably available in electronic medical records. Therefore, these variables could not be included in the analyses, which may have limited a more comprehensive evaluation of factors associated with prolonged ICU stay. Finally, only a type IV MiECC system was evaluated in this study, though different MiECC configurations may produce different clinical outcomes.

## 5. Conclusions

The aim of this study was to identify the preoperative predictors of prolonged ICU stay following isolated coronary artery bypass grafting, with a specific focus on the potential protective role of minimally invasive extracorporeal circulation systems. Although MiECC is theoretically advantageous in reducing systemic inflammation and promoting faster postoperative recovery, our study did not demonstrate a statistically significant association between the utilization of MiECC and a reduction in ICU duration. Conversely, lower preoperative ejection fraction and elevated baseline leukocyte count were independently associated with prolonged ICU stay. These findings suggest that baseline cardiac reserve and preoperative inflammatory status may contribute to postoperative recovery. Although no independent association between MiECC utilization and prolonged ICU stay could be demonstrated, the possibility of a modest protective effect cannot be completely excluded given the limited sample size and wide confidence intervals observed in the present study. These findings should be regarded as hypothesis-generating and require validation in future large-scale prospective studies to better define the specific high-risk patient subgroups that may truly benefit from MiECC.

## Figures and Tables

**Table 1:** Comparison of patient characteristics and laboratory findings based on ICU stay duration.

	Prolonged ICU Stay	Non-Prolonged ICU Stay	*p*-Value
	(n = 32)	(n = 50)	
Gender			0.363 ^+^
Male	25 (78.1)	36 (72)	
Female	7 (21.9)	14 (28)	
Diabetes Mellitus	21 (65.6)	30 (60)	0.392 ^+^
Hypertension	25 (78.1)	37 (74)	0.440 ^+^
COPD (Chronic Obstructive Pulmonary Disease)	2 (6.3)	2 (4)	0.510 ^+^
Hyperlipidemia	15 (46.9)	28 (56)	0.420 ^+^
CKD (Chronic Kidney Disease)	0 (0)	2 (4)	0.369 ^+^
Age	64 ± 11.2	63.75 ± 9.08	0.805 *
Body Surface Area (BSA)	1.84 ± 0.24	1.83 ± 0.2	0.779 *
Ejection Fraction (%)	52.5 (19)	60 (5)	0.012 ^#^
Hemoglobin (g/dL)	13.8 (2.32)	13.4 (2.32)	0.222 ^#^
Hematocrit (%)	42.1 (7.17)	40.05 (6.88)	0.260 ^#^
Neutrophils (10^3^/µL)	5.11 (3.09)	4.65 (1.55)	0.139 ^#^
Leukocytes (10^3^/µL)	8.4 (4.25)	7.97 (2.03)	0.049 ^#^
Platelets (10^3^/µL)	263 (102)	237.5 (116.75)	0.488 ^#^
AST (U/L)	24 (12.5)	20 (10)	0.311 ^#^
ALT (U/L)	19.5 (10.75)	20 (15.75)	0.498 ^#^
Urea (mg/dL)	39.3 (16.1)	34.1 (16.25)	0.285 ^#^
Creatinine (mg/dL)	0.92 (0.27)	0.87 (0.32)	0.316 ^#^
Sodium (mmol/L)	139 (5.55)	138.2 (3.5)	0.668 ^#^
Potassium (mmol/L)	4.53 (0.67)	4.33 (0.56)	0.300 ^#^
Albumin (g/L)	43.05 (3.33)	41.1 (5.42)	0.215 ^#^
CRP (mg/L)	6.51 (13.61)	4.69 (12.77)	0.992 ^#^
HbA1c (%)	6.75 (2.55)	6.45 (2.55)	0.489 ^#^

^+^: Chi-square Test, *: Independent Samples *t*-test, ^#^: Mann–Whitney U Test. COPD: Chronic Obstructive Pulmonary Disease, CKD: Chronic Kidney Disease, BSA: Body Surface Area, AST: Aspartate Aminotransferase, ALT: Alanine Aminotransferase, CRP: C-Reactive Protein, HbA1c: Glycated Hemoglobin.

**Table 2:** Comparison of intraoperative parameters according to ICU stay duration.

	Prolonged ICU Stay	Non-Prolonged ICU Stay	*p*-Value
	(n = 32)	(n = 50)	
CPB Type			0.530 ^+^
MiECC	9 (28.1)	15 (30)	
Conventional	23 (71.9)	35 (70)	
Cross-Clamp Time (min)	75 (48)	60 (29)	0.099 ^#^
CPB Time (min)	98 (73)	93 (50)	0.161 ^#^
Number of Bypasses	3.5 (1)	3 (1)	0.173 ^#^

^+^: Chi-square test, ^#^: Mann–Whitney U test. MiECC: minimally invasive extracorporeal circulation, CPB: cardiopulmonary bypass.

**Table 3:** Univariate logistic regression analyses for predictors of prolonged ICU stay.

	Univariate OR (%95 CI)	*p*
Age	1.003 (0.959–1.050)	0.885
Diabetes mellitus (Ref.: No)	1.273 (0.506–3.204)	0.609
COPD (Ref.: No)	1.600 (0.214–11.969)	0.647
New-onset atrial fibrillation (Ref.: No)	1.708 (0.657–4.437)	0.272
Acute kidney injury (Ref.: No)	1.212 (0.378–3.888)	0.747
CPB time	1.008 (0.998–1.018)	0.122
Number of bypass grafts	1.385 (0.900–2.133)	0.139
Cross-clamp time	1.013 (0.999–1.026)	0.064
Ejection fraction (%)	0.935 (0.889–0.984)	0.010
White blood cell count (10^3^/µL)	1.382 (1.092–1.749)	0.007
C-reactive protein (mg/L)	0.998 (0.983–1.013)	0.788
MiECC (Ref.: No)	0.913 (0.343–2.432)	0.856

CPB: cardiopulmonary bypass, COPD: chronic obstructive pulmonary disease, MiECC: minimally invasive extracorporeal circulation, OR: odds ratio, CI: confidence interval.

**Table 4:** Multivariate logistic regression models for independent predictors of prolonged ICU stay.

	Multivariate OR (%95 CI)	*p*
Model 1		
Age	1.015 (0.963–1.069)	0.587
Ejection Fraction (%)	0.938 (0.889–0.990)	0.021
White Blood Cell Count (10^3^/µL)	1.351 (1.052–1.733)	0.018
MiECC Utilization (Ref.: No)	0.727 (0.242–2.187)	0.570
Model 2		
Age	1.022 (0.968–1.079)	0.433
Ejection Fraction (%)	0.948 (0.897–1.002)	0.058
White Blood Cell Count (10^3^/µL)	1.359 (1.054–1.752)	0.018
MiECC Utilization (Ref.: No)	0.839 (0.275–2.563)	0.758
Cross-Clamp Time	1.010 (0.995–1.026)	0.175
Model 3		
Age	1.020 (0.967–1.077)	0.464
Ejection Fraction (%)	0.944 (0.894–0.997)	0.038
White Blood Cell Count (10^3^/µL)	1.361 (1.055–1.757)	0.018
MiECC Utilization (Ref.: No)	0.813 (0.267–2.481)	0.716
Cardiopulmonary Bypass Duration	1.006 (0.995–1.017)	0.255
Model 4		
Age	1.008 (0.960–1.058)	0.752
Ejection Fraction (%)	0.928 (0.879–0.980)	0.007
C-Reactive Protein (mg/L)	0.995 (0.978–1.011)	0.526
MiECC Utilization (Ref.: No)	0.683 (0.233–1.997)	0.486

MiECC: minimally invasive extracorporeal circulation, OR: odds ratio, CI: confidence interval; Model 1: χ^2^ = 14.317, *p* = 0.006, Cox and Snell R^2^ = 0.160, Nagelkerke R^2^ = 0.217; Model 2: χ^2^ = 16.468, *p* = 0.006, Cox and Snell R^2^ = 0.182, Nagelkerke R^2^ = 0.247; Model 3: χ^2^ = 15.609, *p* = 0.008, Cox and Snell R^2^ = 0.173, Nagelkerke R^2^ = 0.235; Model 4: χ^2^ = 8.276, *p* = 0.082, Cox and Snell R^2^ = 0.173, Nagelkerke R^2^ = 0.235.

**Table 5:** ROC analysis of ejection fraction, white blood cell count, and multivariate logistic regression models for predicting prolonged ICU stay.

	AUC (%95 CI)	*p*	Cut-Off	Sensitivity (%)	Specificity (%)
Ejection Fraction (%)	0.656 (0.533–0.779)	0.018	≤59.0	65.6	56
White Blood Cell Count (10^3^/µL)	0.627 (0.496–0.757)	0.054	≥8.25	56.3	64
Multivariate Model 1	0.745 (0.634–0.856)	<0.001	---	84.4	52
Multivariate Model 2	0.749 (0.637–0.862)	<0.001	---	84.4	56
Multivariate Model 3	0.743 (0.630–0.856)	<0.001	---	84.4	48
Multivariate Model 4	0.662 (0.537–0.786)	0.014	---	71.9	50

AUC: area under the curve; CI: confidence interval; CRP: C-reactive protein; EF: ejection fraction; MiECC: minimally invasive extracorporeal circulation; WBC: white blood cell count. Model 1: age, EF, WBC, and MiECC utilization. Model 2: age, EF, WBC, MiECC utilization, and cross-clamp time. Model 3: age, EF, WBC, MiECC utilization, and cardiopulmonary bypass duration. Model 4: age, EF, CRP, and MiECC utilization.

## Data Availability

The data used and/or analyzed during the current study are not publicly available due to patient privacy and ethical restrictions, but are available from the corresponding author on reasonable request.

## Abbreviations

The following abbreviations are used in this manuscript:

CABG	Coronary artery bypass grafting
ICU	Intensive care unit
MiECC	Minimally invasive extracorporeal circulation
CPB	Cardiopulmonary bypass
cCPB	Conventional cardiopulmonary bypass
ACT	Activated clotting time
SIRS	Systemic inflammatory response syndrome
COPD	Chronic obstructive pulmonary disease
CKD	Chronic kidney disease
LVEF	Left ventricular ejection fraction
CRP	C-reactive protein
HbA1c	Glycated hemoglobin
LCOS	Low cardiac output syndrome
BSA	Body surface area
CI	Confidence interval
OR	Odds ratio
SD	Standard deviation
IQR	Interquartile range
